# Takayasu Arteritis in the pediatric population: a contemporary United States-Based Single Center Cohort

**DOI:** 10.1186/1546-0096-12-21

**Published:** 2014-06-04

**Authors:** Heidi S Szugye, Andrew S Zeft, Steven J Spalding

**Affiliations:** 1 Cleveland Clinic Children’s, Cleveland, Ohio, USA; 2 Center for Pediatric Rheumatology, Cleveland Clinic Children’s, Cleveland, Ohio, USA; 3 Center of Pediatric Rheumatology, Center for Pediatric Rheumatology, Cleveland Clinic Children’s, 9500 Euclid Avenue/Desk A111, Cleveland, OH 44195, USA

**Keywords:** Takayasu Arteritis, Vasculitis, Children, United States, Cleveland Clinic Children’s Center for Pediatric Rheumatology

## Abstract

**Background:**

Takayasu Arteritis is an idiopathic, chronic, large vessel vasculitis involving the aorta and its primary branches. Few studies have been done in pediatric patients to date with the largest case series of US patients published in 2003 consisting of only 6 patients.

**Methods:**

A retrospective chart review was performed on all patients seen at Cleveland Clinic Children’s up until 2012 who met EULAR/PRINTO/PRES classification criteria for childhood Takayasu Arteritis.

**Results:**

Twenty-one patients with a mean follow up of 2.3 years were studied. Weight loss, fatigue, and anorexia were the most common presenting complaints. 57.1% of patients were hypertensive at first visit. The most common examintation finding was diminished pulses (61.9%), followed by bruits, and then murmurs. Thoracic aorta stenosis was the most common vascular abnormality. Seven of twenty-one patients responded well to methotrexate and prednisone alone. Ten of twenty-one patients required an additional medication for symptom and disease control (infliximab most commonly). About two-thirds of patients required at least one anti-hypertensive medication. Eight of the twenty-one patients required surgical intervention for severe disease refractory to medications (renal artery stenosis being the most common indication). Almost all patients reported symptomatic improvement after surgical intervention. Two of the eight patients required a second surgery for return of symptoms. Disease sequelae included arterial aneurysms, resolved heart failure, and hypertensive emergencies.

**Conclusion:**

Our study emphasizes that constitutional symptoms coupled with objective findings of diminished pulses, bruits, and hypertension should raise clinical suspicion for Takayasu Arteritis in pediatric patients. Pharmacologic therapy alone can be successful in controlling disease progression, however surgery was successful in minimizing symptoms when medical therapies failed.

## Background

Takayasu Arteritis (TA) is an idiopathic, chronic, large vessel arteritis involving the aorta and its primary branches [1]. The pathologic course of the disease begins with panarteritis in the adventitia which progresses to the intima, eventually causing vascular narrowing, occlusion, and later in disease, aneurismal formation [2]. The incidence of TA in the pediatric population is unknown but has been estimated at 2.6/1,000,000 over all age groups [3].

Few studies have been performed in pediatric TA patients to date. An article published by Brunner et al. in 2010 summarized the pertinent findings from almost all studies in pediatric TA patients to date including 9 case series from medical centers in Turkey, Canada, USA, South Africa, Korea, India, and Mexico and 41 case reports representing a total of 241 patients. The largest case series of US patients published in 2003 included only 6 patients [4].

TA typically manifests with an initial acute phase of constitutional symptoms which correlate with the pathologic inflammatory vascular changes and progresses to a second phase with symptoms of claudication and hypertension and findings of pulselessness and bruits suggestive of arterial occlusion and ischemia [5]. If diagnosis is delayed, inflammation can progress to involve more areas of vasculature and lead to stenosis, aneurysms, and eventually end-organ ischemia. Unfortunately, one-third of children present after the acute phase and suffer effects of tissue ischemia with a median time from symptom onset to diagnosis of 19 months, close to four times longer than adults [3,6,7].

Pediatric TA is largely a clinical diagnosis, currently based on the European League Against Rheumatism (EULAR)/Pediatric Rheumatology International Trials Organization (PRINTO)/Pediatric Rheumatology European Society (PRES) criteria [8].

To date, no comparative therapeutic studies have been conducted in pediatric TA. Treatment options for TA are broad and variations in care abound. Medical therapy often starts with glucocorticoids and methotrexate although recently other immunosuppressants and TNF inhibitors have had reported efficacy with disease suppression. When a patient’s disease remains active or is deemed “severe”, physicians have also employed alkylating agents such as cyclophosphamide. Surgical interventions are used when severe stenosis is present and threatening organ perfusion but minimal evidence of indications, outcomes, and sustainability of treatment exist.

The primary aim of this study was to review the clinical presentation of TA in a contemporary cohort of United States children using recently validated EULAR/PRINTO/PRES classification criteria, including the frequencies of anatomic arterial involvement. The secondary aim was to document treatment choices and outcomes in this pediatric cohort.

## Methods

This retrospective cohort study was approved by the Cleveland Clinic Institutional Review Board which waived patient consent. A search of the Cleveland Clinic (CC) electronic medical record system was performed up until 2012 to identify patients diagnosed at 18 years of age or younger at Cleveland Clinic Children’s (a quaternary referral center) with an International Classification of Diseases, Ninth Revision code of Takayasu Arteritis (446.7). Of the 28 patients labeled with a diagnosis of Takayasu Arteritis, 21 of these patients fulfilled EULAR/PRINTO/PRES criteria for childhood TA (Table 1) [8]. The initial 2006 EULARPRES criteria stated a diagnosis of childhood TA is made when there exists evidence of angiographic abnormalities plus the presence of at least one of the following: decreased peripheral artery pulse (s) or claudication of extremities, a blood pressure difference of >10 mm Hg, bruits over the aorta or its major vessels, or hypertension (related to childhood normative data) [9]. In 2010, these criteria were revised after reviewing clinical features of 99 patients with TA. The revised EULAR/PRINTO/PRES criteria stated the angiographic findings could include either conventional MRI or CT findings, blood pressure discrepancy could be present in any two limbs, and hypertension was defined as systolic or diastolic blood pressure >95th percentile for height. Also, ESR >20 mm/hr or abnormal CRP was added as an additional supporting criteria. With these new additions, the study reported 100.0% sensitivity and 99.9% specificity [8]. Patients were excluded if they were >18 years of age at time of diagnosis (none) or did not meet the EULAR/PRINTO/PRES criteria (7 patients).

**Table 1:** Percentage of patients who fulfilled EULAR/PRINTO/PRES criteria for childhood Takayasu Arteritis

**Criteria**	**No & (%) of patients**
Presence of Angiographic (conventional, MRI, or CT) Abnormalities	21/21 (100.0)
1. Decreased Peripheral Artery Pulse (s) or Claudication of Extremities (Any visit)	16/21 (76.2)
2. Blood Pressure Difference of >10 mm Hg between any two limbs (Based on 1st visit BP)	15/18 (83.3)
3. Bruits Over Aorta or Its Major Vessels (Any visit)	12/21 (57.1)
4. Hypertension (>95th percentile based on presentation or 1st visit BP)	12/21 (57.1)
5. Elevated ESR (>20 mm/hr)	16/21 (76.2)
Met 1 supporting criteria	2/21 (9.5)
Met 2 supporting criteria	5/21 (23.8)
Met 3 supporting criteria	5/21 (23.8)
Met 4 supporting criteria	4/21 (19.0)
Met 5 supporting criteria	5/21 (23.8)

Supporting criteria = The criteria besides angiographic abnormalities (1–5).

Data collected included the patient’s age, gender, race/ethnicity, family history of any rheumatologic conditions, pertinent past medical history, and duration of care at Cleveland Clinic Children’s. Clinical data collected included date of symptom onset, presenting symptoms, and symptoms present throughout care. Vital signs collected included four extremity blood pressures, weight, weight for age (per CDC growth charts), height, and BMI percentile at first visit. Physical examination findings collected included documentation of diminished pulses, bruits, murmurs, rashes, muscle weakness, and ophthalmologic abnormalities. Laboratory data collected was white blood cell count, platelet count, hemoglobin, ESR, CRP, creatinine, albumin, and cholesterol levels at initial presentation and last visit. Vascular study findings collected included results from the following imaging modalities: vascular duplex studies, magnetic resonance angiogram (MRA), computed tomography angiogram (CTA), arteriogram, and echocardiogram with attention to arterial location of involvement and type of lesion present. Therapeutic data collected included medications at time of first visit and throughout care. Indications for surgical interventions along with type of surgery and outcomes were reviewed and collected. Adverse events and disease sequelae were evaluated and collected.

Data was stored in RedCap. Qualitative statistical analysis of the data was performed using Microsoft Excel software.

## Results

### Patient demographics

Seventy-one percent (15) of the 21 patients were female (3.2:1 female to male ratio). Fifty-two percent of patients were of Caucasian race. 3 patients were Hispanic, 1 patient was from Jordan, 1 patient was African American, 1 patient was American Indian, 1 patient was Asian, and 1 patient was from Israel. Median duration of care at CC was 1.2 years (1 day - 7.6 years) with a mean of 2.3 ± 2.5 years with 71.4% of patients having more than one outpatient rheumatology visit at CC. The earliest first visit date was in 2003 and eight of twelve patients had been seen since 2011. Concomitant medical diagnoses in TA patients included iron deficiency anemia (n = 4), inflammatory bowel disease (n = 2), migraines (n = 2), ADHD (n = 2), pyoderma gangrenosum (n = 1), and thyroid papillary carcinoma (n = 1). One patient had a history of a positive purified protein derivative (PPD) test about 9 years prior to onset of symptoms for which he was treated with isoniazid. None of the patients had family members with any vasculitides. Frequency of autoimmune disease in extended family members was 4/21 and included celiac disease, mixed connective tissue disease, juvenile idiopathic arthritis, and systemic lupus erythematosus.

### Clinical features

The median age of symptom onset was 13 years (1.5 months - 17 years) with a mean of 11.5 ± 4.4 years. The median time from symptom onset to diagnosis was 6 months (1 month - 14 years) with a mean of 19.3 ± 35.4 months. All symptoms and their frequencies at disease onset and throughout care are listed in Table 2. Weight loss (47.6%), fatigue (38.0%), anorexia (23.8%), and dyspnea (19.1%) were the most common presenting complaints. Fatigue (28.6%), fever (23.8%), dyspnea (19.1%), paresthesias (19.1%), and weight loss (19.1%) were the most common complaints after initial diagnosis.

**Table 2:** Symptoms at presentation and throughout disease in children with Takayasu Arteritis

**Symptom**	**Patients experiencing symptom at disease presentation (our study)**	**Patients experiencing symptom throughout follow up care (our study)**	**Patients experiencing symptom at disease presentation or throughout care (Brunner et al. Review)**[6]
	**(n = 21)**	**(n = 21)**	**(n = 160-230) (%)**
**Anorexia**	5 (23.8)	1 (4.8)	NR
**Arthralgias**	3 (14.3)	3 (14.3)	33/230 (14.3)
**Back Pain**	3 (14.3)	3 (14.3)	NR
**Blurred Vision**	2 (9.5)	2 (9.5)	NR
**Carotidynia**	1 (4.8)	1 (4.8)	NR
**Chest Pain**	1 (4.8)	3 (14.3)	15/199 (7.5)
**Dizziness**	0 (0.0)	1 (4.8)	NR
**Dyspnea**	4 (19.1)	4 (19.1)	49/210 (23.3)
**Emesis**	2 (9.5)	0 (0.0)	40/199 (20.1)
**Fatigue**	8 (38.0)	6 (28.6)	NR
**Fever**	3 (14.3)	5 (23.8)	47/160 (29.4)
**Headache**	3 (14.3)	3 (14.3)	66/210 (31.4)
**Jaw Pain**	2 (9.5)	0 (0.0)	NR
**Lower Extremity Claudication**	3 (14.3)	3 (14.3)	NR
**Myalgias**	0 (0.0)	3 (14.3)	NR
**Night Sweats**	3 (14.3)	1 (4.8)	NR
**Non- Post-Prandial Abdominal Pain**	1 (4.8)	2 (9.5)	33/199 (16.6)
**Palpitations**	1 (4.8)	1 (4.8)	29/199 (14.6)
**Paresthesias**	1 (4.8)	4 (19.1)	NR
**Post-Prandial Abdominal Pain**	1 (4.8)	2 (9.5)	NR
**Rash/Skin Lesions**	0 (0.0)	2 (9.5)	12/230 (5.2)
**Seizure-like Activity**	2 (9.5)	0 (0.0)	NR
**Syncope**	1 (4.8)	2 (9.5)	4/199 (2.0)
**Upper Extremity Claudication**	0 (0.0)	3 (14.3)	NR
**Weight Loss**	10 (47.6)	4 (19.1)	NR

NR = Not Reported.

Of the recorded blood pressures at onset or first visit, 15 of the 18 patients had a >10 mm Hg difference in systolic blood pressure between any two limbs, and 12 patients (57.1%) were classified as being hypertensive with systolic blood pressures >95th percentile for age, sex, and height (Table 1).

Abnormal examination findings throughout disease were reviewed. Thirteen of 21 (61.9%) patients had diminished pulses on examination. Of these, 6 had diminished radial pulse (s), 5 diminished dorsalis pedis pulse (s), 4 diminished carotid pulse (s), 3 diminished femoral pulse (s), and 2 diminished brachial pulse (s). Bruits were heard in 12 of 21 of our patients (57.1%) as noted in Table 1. Of these, 7 had a carotid bruit on examination, 4 a renal bruit, and 3 an aortic bruit. Pathologic sounding murmurs were heard in 6 patients (28.6%). Two patients had muscular weakness. Two patients had erythema nodosum. One patient had posterior vitreous detachment and degeneration per dilated ophthalmologic examination.

### Laboratory findings

Laboratory data at time of presentation and last visit was reviewed when available. Of the ESRs reported on our patients (n = 15) at presentation, 100% had an elevated ESR with a mean of 78.6 mm/hr. Of the ESRs reported at follow up (n = 16), only 37.5% of patients had an elevated ESR. Of the 13 patients for which a presenting ESR and follow up ESR were available, there was a mean decrease in ESR of 66.7%. Only 1 patient had an elevated creatinine; this patient had “abnormal heterogeneous perfusion of the renal parenchyma with pruned appearance of the intraparenchymal vessels” on aortogram.

### Vascular study findings

**Figure 1:**
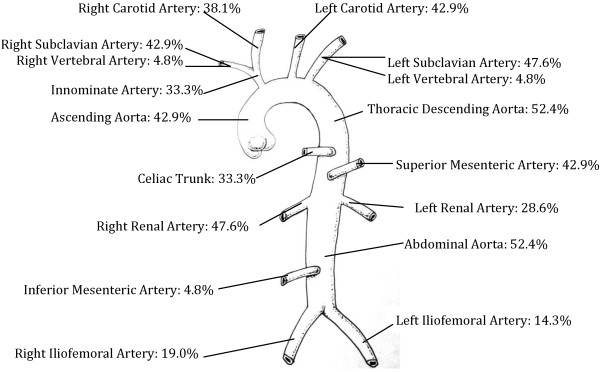
Prevalence of specific arterial involvement in children with Takayasu Arteritis.

**Figure 2:**
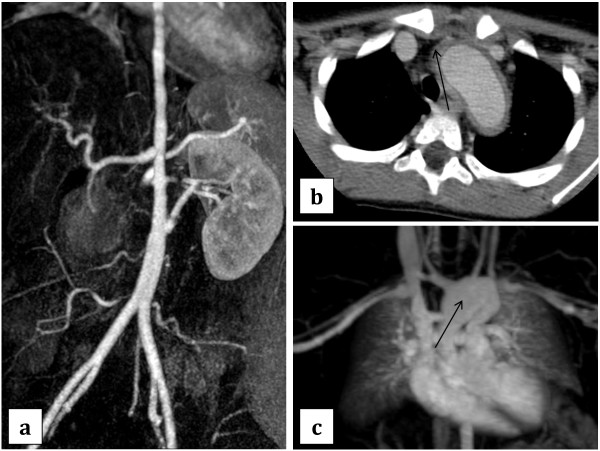
**Radiologic abnormalities in children with Takayasu Arteritis. a**. Patient 4 per Table 4 with MRA showing significantly decreased caliber and irregularity of the descending thoracic and abdominal aorta, with minimally increased signal characteristics, consistent with inflammatory changes, notably in the proximal descending segment. Patient is status post right sided nephrtectomy with single left renal artery, which appears occluded in its mid segment with patent bypass graft arising from the mid infra-renal abdominal aorta to the left kidney. **b**. Patient 7 per Table 4 with CTA showing inflammation around the aorta. **c**. Patient 7 per Table 4 with MRA findings of a mildly prominent proximal ascending aorta, moderately dilated distal ascending aorta, and aneurysmal transverse aortic arch extending into the proximal descending aorta. The largest transverse aortic arch dimension is 30 × 26 mm.

Table 3 lists the number of patients with a specific type of vascular abnormality at different arterial locations. The most common type of abnormality in our patients was stenosis (66.2% of all lesions) followed by edema, occlusion, and inflammation. Figure 1 illustrates the percentage of patients with any type of vascular involvement by anatomic location. The thoracic and abdominal aorta were the most commonly affected locations (52.4% each) followed by the right renal artery (47.6%) and the left subclavian artery (47.6%). Typical MRA and CTA findings are shown in Figure 2.

**Table 3:** Frequency of different arterial abnormalities at specific sites in 21 children with Takayasu Arteritis

**Type of abnormality**	**AA**	**IA**	**LSA**	**RSA**	**LVA**	**RVA**	**LCA**	**RCA**	**TDA**	**CT**	**SMA**	**LRA**	**RRA**	**AbA**	**IMA**	**LIF**	**RIF**	**PA**	**Total**
**Aneursym**	0	0	1	1	0	0	0	0	1	0	0	0	0	1	0	0	0	0	4
**Dilation**	4	1	1	0	0	0	2	0	1	0	0	0	0	0	0	0	0	0	9
**Edema**	2	2	0	1	0	0	1	1	3	0	0	0	0	3	0	0	0	0	13
**Inflammation**	2	0	0	1	0	0	1	1	3	0	0	0	0	2	0	0	0	0	10
Stenosis*	7	6	6	7	1	1	9	8	9	6	5	5	8	9	1	3	4	1	96
**Occlusion**	0	0	0	1	0	0	1	0	0	1	4	1	2	0	0	0	0	0	10
**Plaque**	0	0	0	0	0	0	1	1	0	0	0	0	0	0	0	0	0	0	2
**Thrombus**	0	0	0	0	0	0	0	0	0	0	1	0	0	0	0	0	0	0	1
**Total**	15	9	8	11	1	1	15	11	17	7	10	6	10	15	1	3	4	1	

*Definition for renal artery stenosis = 60% or greater on Duplex.

AA = Ascending Aorta, IA = Innominate Artery, LSA = Left Subclavian Artery, RSA = Right Subclavian Artery, LVA = Left Vertebral Artery, RVA = Right Vertebral Artery, LCA = Left Carotid Artery, RCA = Right Carotid Artery, TDA = Thoracic Descending Aorta, CT = Celiac Trunk, SMA = Superior, Mesenteric Artery, LRA = Left Renal Artery, RRA = Right Renal Artery, AbA = Abdominal Aorta, IMA = Inferior Mesenteric Artery, LIF = Left Iliofemoral Artery, RIF = Right Ileofemoral Artery, PA = Pulmonary Artery.

### Treatment

The majority of our patients were treated with prednisone and methotrexate at some point in their disease course (85.7% and 65.7%, respectively). Infliximab was the next most commonly used medication (42.9%). Indications for adding, increasing dose of, or changing medication included new, worsening, or persistent symptoms, new physical examination findings, elevated inflammatory markers, or worsening findings on imaging studies. Two patients required only prednisone to control signs and symptoms related to TA. Seven of 21 (33.3%) patients clinically responded favorably to methotrexate and prednisone alone. Ten of 21 patients (47.6%) required a medication in addition to prednisone and methotrexate for symptom and disease control [anakinra (n = 3), cyclophosphamide (n = 5), etanercept (n = 2), infliximab (n = 9), IV steroids (n = 6), or mycophenolate mofetil (n = 3)]. Infliximab was the most commonly used medication when disease activity was still present despite prednisone and methotrexate use (9/21 patients [42.9%]). The majority of patients (14/21; 66.7%) required at least one anti-hypertensive medication. Beta blockers were used in almost half our patients to treat hypertension. Aspirin was used in 38.1% of patients when the presence of stenotic lesions warranted thrombosis prevention.

Of the 21 patients followed, 8 (38.1%) required surgical intervention while 13 were managed with medical treatment alone. Table 4 summarizes surgical procedures performed, the indication for the procedure, and post-surgical outcomes for this cohort. The most commonly involved vascular site requiring surgical correction was the renal arteries (5/8 patients) while the most common indication for correction of the renal arteries was hypertension with evidence of severe stenosis on imaging. These patients either underwent angioplasty, stenting, bypass, and/or nephrectomy. The remaining three patients required surgical intervention for ascending aorta and aortic arch involvement, 2 of which had aneurysmal changes. One patient required PA stenting. Two of the 8 patients (patients 4 and 5) failed renal artery angioplasty and required renal artery bypass surgery due to return of symptoms and/or findings on imaging. Both of these patients had severe hypertension requiring intravenous anti-hyptertensives. Patient 6 underwent renal artery stenting; he was found to be asymptomatic and had patent vasculature at follow up. Patient 7 reported symptomatic improvement but no follow up imaging was available. Four of the 8 patients (1, 2, 3, and 8) reported resolution of symptoms but did show some return of vascular stenosis or aneurismal change on post-op imaging; however, vascular changes were not severe enough to warrant a secondary procedure. Patient 3 demonstrated improvement in post-op renal artery stenosis after treatment with methotrexate.

**Table 4:** Outcomes of children who underwent surgical intervention for medication-refractory Takayasu Arteritis

**Patient**	**Surgical procedure (s)**	**Indication (s)**	**Outcome (s)**
**1**	-Ross procedure	-Severe aortic regurgitation on echocardiogram; diplopia; symptoms of heart failure	-Improvement in symptoms; mild aortic narrowing on MRA ~6 months post-op; annulo-aortic ectasia with enlargement of root and ascending aorta per CTA ~2 years post op; mild aortic regurgitation on echocardiogram ~3 years post-op
	-Pulmonary artery stent placement	-Severe pulmonary artery narrowing	-No significant stenosis of the main or central pulmonary arteries ~1 year and ~2 years post op per CTA; diastolic flow reversal in the proximal pulmonary artery branches suggestive of free pulmonary insufficiency on echocardiogram ~2 years post-op
**2**	-Replacement of ascending aorta, aortic arch, and proximal descending aorta with elephant trunk procedure in the descending aorta	-Severe lower extremity claudication, saccular aneurysm of the distal arch and aortic isthmus per MRA	-No improvement of lower extremity claudication; stable appearance of graft ~6 months post op
	-Abdominal aortic aneurysm resection, infrarenal graft, and IMA revascularization	-SMA occlusion, infrarenal stenosis per MRA	-Resolution of lower extremity claudciation ~1 year post op; intact infrarenal graft, proximal superior SMA occlusion, reimplanted inferior mesenteric artery appears moderately narrowed at the anastomosis on MRA ~ 1 year post op
**3**	-Right renal artery angioplasty	-Severe hyptertension; headache; severe right renal artery stenosis per MRA	-Improvement in hypertension and headaches; right renal artery stenosis per MRA and ultrasound ~2 months post op; widely patent right renal artery ~4 years post op after medicinal treatment with methotrexate
**4**	-Bilateral renal artery angioplasty	-Malignant hypertension requiring IV anti-hypertensive drip	-Initial improvement of blood pressure, but 9 days later developed worsening hypertension and fatigue
	-Right nephrectomy and left kidney aorto-renal bypass with the left saphenous vein	-Severe hypertension again requiring IV anti-hypertensive drip and fatigue	-Improved hypertension; Mild stenosis of left renal artery 3 days post op per renal ultrasound; MRA 5 days post op with left renal artery occlusion in its mid segment with patent bypass graft
**5**	-Renal artery angioplasty	-Renal arteries and SMA occlusion per CTA	-Improved hypertension
	-Right and left renal arteries and SMA bypass	-Severe hypertension again requiring IV anti-hypertensive drip; bilateral severe renal artery stenosis on CTA	-Further improvement of hypertension; MRA ~1 week post op with patent SMA graft with occlusion at ostium, patent right renal artery, patent left renal artery bypass with occlusion at ostium; ~1 year post op CTA with patent left renal artery bypass, mild stenosis of right renal artery, SMA occluded but asymptomatic
**6**	-Left renal artery stent	-Severe left renal artery stenosis per CTA; hypertenstion	-Improvement in hypertension; MRA ~6 months post-op with patent bilateral renal arteries; patent on MRA ~3 years post op
**7**	-Right nephrectomy	-Severe renal artery stenosis; hypertension	-Improvement of hypertension
**8**	-Replacement of aortic arch and ascending aorta	-Aneurysmal dilatation of the ascending thoracic aorta, complete occlusion of the proximal left subclavian artery per MRA	-2 days post op CTA showed intact graft; MRA ~1 year post op with stable aortic dimensions; MRA ~3 years post op with thickening to the aortic arch and interval development of thickening and dilation of the descending aorta; patient has remained on infliximab since surgery

### Disease sequelae

Four patients had findings of arterial aneurysms on imaging. One patient suffered ophthalmologic damage. One patient suffered symptoms of heart failure and acute tubular necrosis just prior to receiving her Ross procedure; these symptoms resolved following surgical interventions. Three patients suffered from hypertensive emergency. Two patients required admission to a PICU for IV anti-hypertensive drips to control hypertension prior to renal artery stenting. Steroid adverse effects were seen in many of our patients including steroid-induced Cushingoid features (42.9%) and decreased bone density (3 patients). Psychological diagnoses, including anxiety and depression, were found in 3 patients. None of our patients to our knowledge have suffered any of the following potential complications of the disease: myocardial infarction, stroke, or death.

## Discussion

TA is a rare large vessel vasculitis and there are few pediatric studies published to date from which to compare with our study results. In our contemporary cohort there is a female predominance similar to other studies [6]. Also, both the mean and median ages of symptom onset (11.5 ± 4.4 and 13 years respectively) were similar to the data presented by Brunner in her review article [6]. Only one of our patients had a positive PPD test, while studies from more endemic regions have reported rates ranging from 29% to 90% [2,10,11]. Interestingly, 1 of our patients and 2 patients in Hahn’s review of 31 patients from South Africa reported a co-diagnosis of papillary thyroid cancer although both had been exposed to total lymphoid irradiation [10]. Inflammatory bowel disease has also been reported multiple times (37 per Kusunoki’s literature review) in patients with TA and also in 2 patients in our study [3,12]. This is possibly related to a genetically-mediated increased propensity for autoimmune-mediated disease processes.

Constitutional symptoms have been reported to be twice as common in pediatric patients compared with adult patients [6]. While the most common symptoms on presentation in our patients were weight loss, fatigue, and anorexia, other series have reported headaches (31.0%), fever (29.0%), and dyspnea (23.0%) to be the most common symptomatic manifestations of TA in children [6]. This discrepancy could be due to a smaller sample size, recall bias of the patient, or possibly these patients being diagnosed earlier as constitutional symptoms manifest earlier in the disease process when there is vasculitis and not necessarily vascular sequelae yet. Symptoms more unique to TA such as carotidynia and claudication were not commonly reported. This could possibly be because they are less frequently assessed symptoms or difficult to describe for patients. Asking about these more rare symptoms could be a clue to diagnosis for clinicians.

Bruits were heard in 12/21 of our patients (57.1%) and were reported in only 38/230 (16.5%) of patients in Brunner’s review of all pediatric patients. Thirteen of 21 (61.9%) patients had diminished pulses on examination which is in contrast with lower rates described by Brunner et al. in which 30/230 patients (13.0%) were found to have poor pulses anywhere; frequency reported in adults is 22.5% [6]. In our patient population, hypertension was the most common objective clinical finding, followed by diminished pulses, and bruits; all were seen in over half of our patients. This is an important finding which highlights the need for thorough and consistent clinical examination including auscultation for bruits in the child with hypertension or otherwise generic constitutional complaints such as weight loss, fatigue, headaches and dyspnea as a means to hasten diagnosis. Of the ESR values reported on our patients, at presentation 100% had an elevated ESR with a mean of 78.6. Although ESR is a very non-specific marker of inflammation, it may be an important test to aid in making an earlier diagnosis when patients present with vague constitutional symptoms. Repeat ESRs may be helpful to follow as a disease activity marker and its lack of normalization should prompt further investigation.

Vascular involvement was assessed primarily by MRA and CTA in our patients. Both can delineate stenosis, dilation, and aneursyms, but MRA may overestimate partial stenosis as complete occlusion [13]. Our patients most commonly had vascular abnormalities involving the thoracic and abdominal aorta, consistent with other studies in pediatric patients [6,13,14]. However, in Mexico and Turkey, pediatric patients more commonly had renal, subclavian, and carotid involvement [11,15]. Genetics or differences in environmental exposures or triggers may play a part in these differences. Similar to other studies, the most common type of lesion in our patients was stenosis followed by occlusion and radiographic signs concerning for inflammation [16,17]. Aneurysms were reported in 19% of our patients while other studies have reported incidences ranging from 41-65% [14]. Aneurysms were found in the right and left subclavian arteries, thoracic descending aorta, and abdominal aorta whereas other studies have shown almost exclusive involvement of the abdominal aorta [14].

In general, a higher percentage of our patients met the supporting criteria per the EULAR/PRINTO/PRES classification compared to the frequencies reported in other studies [6]. All of our patients met at least one supporting criteria, and five patients met all five criteria. While not constructed to be used as a screening tool, these criteria could be used to identify a select group of patients who require additional diagnostic imaging. While symptoms on presentation commonly are non-specific and cannot stand alone as diagnostic criteria, constitutional symptoms should be a red flag to the primary care physician or pediatric rheumatologist. Although less specific and not part of existing diagnostic criteria, from our series there were three patients with cardiac murmurs on examination without diminished pulses. Our data support the added diagnostic utility of elevated ESR. Serum cytokine measures may in the future aid in earlier diagnosis, disease activity assessment, and treatment related decisions as IL-18 and IL-6 have been reported to be higher in adults with TA [18].

There is limited data regarding treatment efficacy outcomes for children with TA. In the adult population, about 60% of patients respond to glucocorticoids alone. This is very different from our findings; only 2 of our patients responded to steroids. This could be due to a more inherently severe disease course in the pediatric population, delayed diagnosis in the pediatric population, or the reluctance of providers treating pediatric onset patients to use systemic corticosteroids as a sole maintenance medication [6]. Studies have shown glucocorticoid therapy alone can consistently induce remission, but time in remission is shortened [3]. Most of our patients who did not respond to steroids and methotrexate were started on infliximab. Severely ill patients were treated with cyclophosphamide. In a limited series of 6 patients, Ozen has shown induction with cyclophosphamide and steroids followed by methotrexate is an effective regimen for pediatric patients with widespread disease [19]. Cyclophosphamide is used sparingly in the adult population due to its potential teratogenic effects so data is limited; however, it may be of great therapeutic use in the pediatric population.

Antiplatelet therapy has also been hypothesized to be beneficial in patients with TA [20]. Plasma levels of markers of thrombosis includingplatelet factor 4, beta-thromboglobulin, thrombin-anti-thrombin III, fibrinopeptide A, and D-dimer are substantially higher in TA patients [21]. This is important since inflammation causes platelet, leukocyte, and endothelial cell activation which leads to a hypercoaguable state [20]. Additionally, many TA patients are on steroids which can have adverse effects on hyperlipidemia, obesity, and hypertension, further increasing the risk of acute ischemic events. In theory, treatment of the underlying inflammation and selection of an effective steroid-sparing or steroid-limiting agent should help alleviate this risk. However, levels of these pro-thrombotic markers have not been different between the active and inactive stages of the disease [20,21]. This suggests treatment with anti-platelet medications such as aspirin, used in 38% of our patients, is prudent. Adult TA patients prescribed anti-platelet medications have shown a decreased incidence of acute ischemic events [22].

Similar to other studies, surgical correction of renal artery stenosis was the most common indication for surgical intervention in our patients [15]. Almost all of our patients reported symptomatic improvement after surgical intervention. Other pediatric studies have shown successful rates of PTA ranging from 83-93% [23,24]. Surgical treatment of any kind was successful in 10 of 12 patients in another study [25]. A 20 year longitudinal study has shown restenosis rates of 18% 5 years after surgery, 5% between the 6th and 10th post-operative years, and 10% between the 11th and 20th years [26]. Reddy et al. reported significant restonsis requiring a second surgery in 20% of patients [25]. Numan’s study suggests stenosis is more likely to recur if patients had greater than 20% stenosis preoperatively and long eccentric lesions. Both of our patients who required a second procedure for renal artery stenosis underwent their second surgery within weeks of their first procedure. Of note, serial angiographic studies have shown that new lesions can be found in 61% of post-surgical patients without symptomatic manifestations [3]. For this reason, routine surveillance is favored to ensure patients are receiving proper medicinal treatment at the onset of new lesions which may prevent the need for surgical interventions. Of note many of our patients with symptomatic improvement post-operatively, developed new lesions found on routine follow up imaging.

No patients died in our study, but the longest duration of follow up was only 8 years. Hahn’s South African Study reported a mortality rate of 22% over 15 years, while the highest mortality rate reported in the recent pediatric literature has been 35% over a 5 year period [11]. These pediatric TA patients were treated solely with glucocorticoids and surgical interventions, suggesting that the use of disease modifying and biologic medications such as methotrexate and infliximab are associated with a decreased rate of mortality [11].

Our study is limited in that it is a retrospective chart analysis of a cohort of children presenting to a quarternary referral center. As such, most of the patients in our study did not initially present to our facility and data at disease onset was sometimes limited if prior medical records were not available for review. Additionally, some patients were not seen on a regular basis at our facility, limiting longitudinal data acquisition. Lastly, all of our patients were adolescents at time of first visit, further limiting the duration of care by a pediatric rheumatologist.

## Conclusion

In conclusion, our study emphasizes the finding that constitutional symptoms as presenting complaints and examination findings of diminished pulses, bruits, and hypertension should raise clinical suspicion for TA. Medical treatment alone can be successful in controlling disease progression, however surgical interventions are successful in eliminating symptoms when medical therapies fail. Further research should be aimed at developing earlier, more specific markers of disease, conducting prospective, randomized controlled studies comparing different therapeutic regimens, and larger, long-term, observational studies examining post-surgical outcomes to improve the outcomes for children with this rare and potentially life threatening disease.

## Abbreviations

TA: Takayasu Arteritis; CC: Cleveland Clinic; EULAR: European League Against Rheumatism; PRINTO: Pediatric Rheumatology International Trials Organization; PRES: Pediatric Rheumatology European Society; MRA: Magnetic resonance angiogram; CTA: Computed tomography angiogram.

## Competing interests

The authors declare that they have no competing interests.

## Authors’ contributions

All authors contributed extensively to the work presented in this paper. HS contributed to the study design, data acquisition and analysis, and manuscript drafting and revisions. AZ assisted with data interpretation and provided critical manuscript revisions. SS contributed to the conception and design of the study, data analysis, and manuscript drafting and revisions. All authors read and approved the final manuscript.
